# Clinical impact of low bone mineral density in patients with colorectal cancer liver metastasis undergoing hepatectomy

**DOI:** 10.1371/journal.pone.0324719

**Published:** 2025-06-23

**Authors:** Yuichi Aoki, Atsushi Miki, Yasunaru Sakuma, Jun Watanabe, Takehiro Kagaya, Makiko Tahara, Takumi Teratani, Kazuhiro Endo, Hideki Sasanuma, Wataru Nishimura, Hisanaga Horie, Joji Kitayama, Naohiro Sata, Hironori Yamaguchi

**Affiliations:** 1 Department of Surgery, Division of Gastroenterological, General and Transplant Surgery, Jichi Medical University, Yakushiji, Shimotsuke, Tochigi, Japan; 2 Division of Cell Biology and Anatomy, Jichi Medical University, Yakushiji, Shimotsuke, Tochigi, Japan; Ehime University Graduate School of Medicine, JAPAN

## Abstract

**Background:**

This study aimed to elucidate the clinical impact of osteopenia on the recurrence of colon cancer liver metastases.

**Methods:**

Patients with colon cancer liver metastases (N = 186) undergoing hepatectomy at Jichi Medical University Hospital between March 2006 and March 2020 were examined retrospectively. Computed tomography (CT) scans on the 11th vertebra within 3 months of surgery assessed bone mineral density (BMD). Age-adjusted BMD determined osteopenia presence. Kaplan-Meier method with a log-rank test estimated survival. Factors associated with survival were assessed using Cox’s proportional hazards model after adjustment for confounders.

**Results:**

Patients with osteopenia had shorter overall (*p* = 0.0001; 5-year overall survival, 51.8% vs 81.8%) and recurrence-free survival (*p* = 0.0008, 5-year recurrence-free survival: 26.3% vs 51.5%) than BMD-normal patients. In multivariable analysis, the risk factor for overall survival was osteopenia (Hazard ratio (HR) 3.79, 95% confidence interval (CI) 2.09–6.87, *p* = 0.001). Risk factors for recurrence were chemotherapy (HR 1.92, 95%CI 1.12–3.30, *p* = 0.002), tumor number (HR 1.51, 95%CI 1.02–2.27, *p* = 0.04), and osteopenia (HR 2.18, 95%CI 1.46–3.24 *p* = 0.001). Patients with osteopenia are more likely to develop lung metastases, and BMD-value reduction associated with KRAS mutation.

**Conclusion:**

Osteopenia may have prognostic significance in patients with liver metastases colorectal cancer.

## Introduction

Colorectal cancer is the third most common cancer and the fourth leading cancer cause of death in the world [1]. More than half of patients develop distant metastases, with the liver or lung being the main metastases sites. Recent advances in chemotherapy and medical technology have improved outcomes, but surgical resection is still the most effective therapy for solitary metastatic colorectal liver cancer. Historically, indications for liver metastases resection have been determined by factors associated with tumor staging such as number, size, and distribution within the liver [2]. Five-year survival rates after resection ranged from 24%−58%, and surgical mortality rates were generally less than 5% [3–5].

Many factors may influence post-liver resection outcomes such as age, gender, race, comorbidities, primary tumor location, primary tumor size, high carcinoembryonic antigen, TNM staging, the extent of distant metastasis, preoperative or postoperative chemotherapy, and radiotherapy [6]. Identifying clinical factors is important for developing an optimal treatment plan, assessing outcomes, and improving survival.

Sarcopenia is defined as reduced skeletal muscle mass and function and has been associated with patient frailty [7]. The European Working Group on Sarcopenia in Older People (EWGSOP) states that sarcopenia is more likely when muscle weakness appears, when the diagnosis is confirmed with reduced muscle mass and quality, and is considered severe due to loss of physical capacity [8,9]. Prevalence of sarcopenia ranges from 12% to 60% in patients with colorectal cancer [10]. There is growing evidence that sarcopenia and increased intra-abdominal fat (central obesity) are associated with poor outcomes [11]. Although sarcopenia is often measured by the psoas muscle index (PMI), intramuscular adipose tissue content (IMAC) has been shown to be associated with nonalcoholic steatohepatitis severity and express skeletal muscle quality [12]. The clinical significance of IMAC in colorectal cancer liver metastasis patients has been reported [13]. In addition, reduced bone mineral density (BMD) is an important survivorship issue in cancer care that exposes patients to osteoporosis risk and consequent fractures which worsen the quality of life and longevity [14]. Osteopenia -- lower than normal reduction in BMD -- is distinct from osteoporosis and has been associated with the prognosis of hepatobiliary cancers [15–18]. In addition, low BMD-indicated osteopenia was predictive for mortality in patients with colorectal cancer liver metastases undergoing liver resection [11]. However, the basis for the association between osteopenia and post-hepatectomy recurrence is still unknown. The present study aims to evaluate the clinical importance of osteopenia in clinical outcomes.

## Materials and methods

### Patients and management

A total of 186 patients who underwent initial resection of colorectal cancer liver metastases between March 2007 and March 2020 at Jichi Medical University Hospital were included. Hepatectomy was performed on surgically controllable patients who could undergo total resection of metastases within a safe liver resection volume based on preoperative assessment of liver function and other organ involvement, including the primary lesion.

The present study was conducted in accordance with the principles embodied in the Declaration of Helsinki, 2013, and all studies were approved by the ethics committees of the Jichi Medical University (clinical 22–046). We assessed the database for data collection for this study from 26/09/2022–26/12/2024. The need for written, informed consent was waived by the Jichi Medical University Hospital Institutional Ethics Board because of the retrospective design. All data were fully anonymized before you accessed them. Patients with no unresectable extrahepatic metastasis who underwent hepatectomy were included. CT scans were performed within 3 months of pre-hepatectomy. In addition to CT scan BMD measurement, patient demographics, comorbidities, operative parameters, and survival outcomes were assessed. Overall survival (OS) was defined as the period from the date of hepatectomy to the date of death or last contact with the patient (censored). For patients with no postoperative recurrence of disease, recurrence-free survival (RFS) was defined as the date of hepatectomy to first recurrence at any site; RFS data were censored for patients who were alive without tumor recurrence at the last follow-up date or who had died without evidence of tumor recurrence. Post-hepatectomy recurrence of colorectal cancer was defined as any newly detected local, hepatic, pulmonary, or extrahepatic tumor by CT scan, positron emission tomography, or magnetic resonance imaging. For lung recurrence, partial resection or systemic chemotherapy was performed, and tumor resection, radiotherapy, or systemic chemotherapy was selected for local recurrence. Systemic chemotherapy was performed using the protocol of infusional 5-fluorouracil/l-leucovorin with oxaliplatin and/or infusional 5-fluorouracil/l-leucovorin with irinotecan.

### Definitions of osteopenia

BMD of the trabecular bone was calculated as average pixel density within the circle of the mid-vertebral core at the lower 11th thoracic vertebra on the preoperative CT [17]. Age-adjusted BMD was expressed in Hounsfield units (HU) and osteopenia presence was according to the method described by Toshima et al [19].

$\begin{array}{c} BMD\;(HU)\;for\;men=308.82-2.49\times Age\;in\;years \end{array}$

$\begin{array}{c} BMD\;(HU)\;for\;women=311.84-2.41\times Age\;in\;years \end{array}$

### Definition of sarcopenia

Skeletal muscle area was measured by CT imaging with a transverse image at the third lumbar vertebrae (L3) level. The psoas muscle in the L3 region was measured with the manual tracing method [19] and normalized to the patient height expressed as PMI (cm2/m2). The cutoff value of PMI was set as 6.36 for men and 3.92 for women as described by Hamaguchi et al [20]. IMAC was calculated as follows [21]: IMAC = region of interest of the multifidus muscle in HU/region of interest of subcutaneous fat in HU. The cutoff value was determined using a receiver operating characteristic curve: −0.5 for males and −0.44 for females.

### Measurement of neutrophil to lymphocyte ratio, platelet to lymphocyte ratio, and prognostic nutrition index

The neutrophil/lymphocyte ratio (NLR) was measured as the number of neutrophils divided by the number of lymphocytes from preoperative blood test results. The platelet/lymphocyte ratio (PLR) was measured by dividing the platelet count by the lymphocyte count. The NLR and PLR cutoff values were set at 1.93 (area under the curve [AUC] = 0.54) and 254 (AUC = 0.49) respectively using a receiver operating characteristic analysis of OS [22]. The prognostic nutrition index (PNI) was measured with 10 × serum albumin value (g/dl) + 0.005 × lymphocytes (/mm3); less than 40 indicates impaired nutrition [18].

### Statistical analysis

Continuous variables were expressed as means ± standard deviation and categorical variables as numbers. All categorical data were analyzed by Pearson’s chi-square test. Normally distributed values were analyzed by Student’s t-test, with the Mann-Whitney U-test for non-normally distributed values. Patient OS curves with or without osteopenia were constructed using the Kaplan-Meier method, as was the log-rank test for the significance of survival. To compare OS with osteopenia, descriptive statistics, and univariable analysis isolated potential confounders. Hazard ratio (HR) and 95% confidence interval (CI) for overall survival were examined using Cox’s proportional hazard model. Odds ratios and CIs were measured using logistic regression analysis for variables related to post-liver resection recurrence. All statistical analyses were performed using JMP version 17. 2. 0 (SAS Institute Inc., Cary, NC) at *p* < 0.05 significance.

## Results

### Patient characteristics

There were 120 males and 66 females in the patient cohort (age M = 61.6y, Table 1). The BMD mean value was 147 ± 47 HU, and 114 (61%) patients had osteopenia. The PMI mean male value was 5.1 cm2/m2, and female value was 3.5 cm2/m2. IMAC mean value in males was – 0.41 and in females was −0.38. The 5-year recurrence-free survival and overall survival rate after hepatectomy for colorectal cancer liver metastasis were 36.6% and 63.7%. There was a significant difference between normal BMD and osteopenia in age, PMI, PLR, and postoperative chemotherapy.

**Table 1:** Patients characteristics.

	Normal BMD N = 79	Osteopenia N = 107	*p* value
Variables			
Age (y), mean ± SD	57.5 ± 11.3	64.6 ± 9.0	0.0001
Gender (male/female), n	55/24	65/42	0.211
BMI (kg × m^-2^), mean ± SD	23.3 ± 3.5	24.0 ± 3.3	0.151
PMI (cm^2^/m^2^), mean ± SD	5.00 ± 1.8	4.15 ± 1.7	0.001
IMAC, mean ± SD	−0.42 ± 0.1	−0.39 ± 0.2	0.206
Albumin (g/dl), mean ± SD	4.07 ± 0.6	4.10 ± 0.4	0.567
NLR, mean ± SD	2.41 ± 1.2	2.64 ± 1.97	0.261
PLR, mean ± SD	188.1 ± 130.5	147.4 ± 69.2	0.026
PNI, mean ± SD	47.8 ± 6.6	48.2 ± 56.0	0.732
CEA (mg/dl), mean ± SD	23.6 ± 50.7	121.7 ± 570.2	0.129
CA19−9 (IU/ml), mean ± SD	141.5 ± 911.7	120.2 ± 295.4	0.822
Extra hepatic lesion (yes/no), n	75/4	94/13	0.087
Timing of tumor (Synchronous/ Metachronous)	57/22	78/29	0.910
Preoperative chemotherapy (yes/no), n	28/51	42/65	0.596
Postoperative chemotherapy (yes/no), n	22/57	53/54	0.002
Maximum tumor size (mm), mean ± SD	36.4 ± 24.2	38.5 ± 43.6	0.697
Lymph node metastases (yes/no)	48/30	69/36	0.561
R0 resection (yes/no)	65/14	93/14	0.382

BMI: Body mass index, PMI; Psoas muscle index, IMAC: Intramuscular adipose tissue composition, NLR: Neutrophil to lymphocyte ratio, PLR: Platelet to lymphocyte ratio, PNI: Prognostic nutrient index, CEA: carcinoembryonic antigen, CA: Carbohydrate antigen

### Relationship between overall survival and patients’ variables

**Fig 1:**
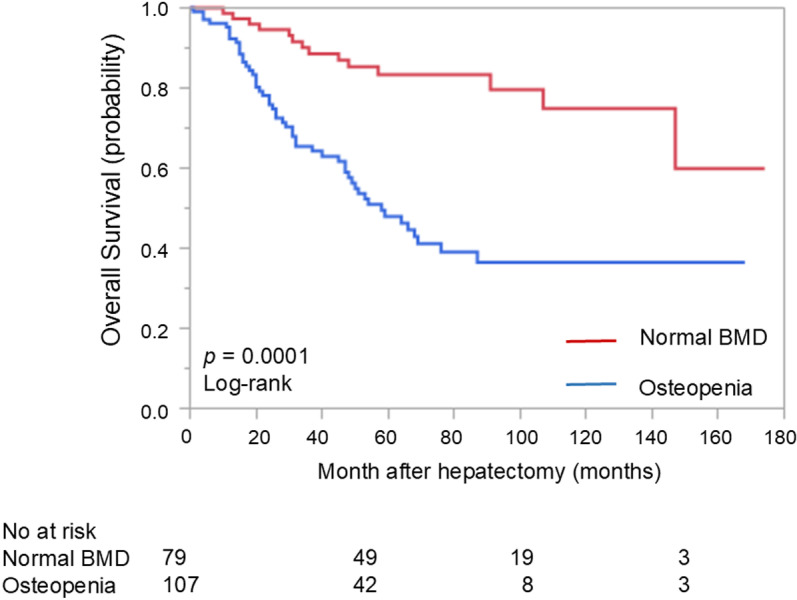
Overall Survival in patients with or without osteopenia.

The median follow-up in the population was 48 months. The OS was significantly shorter in the osteopenia than in normal BMD (*p* = 0.0001; 5-year survival, 51.8% vs 81.8%) (Fig 1).

In univariable analysis for OS, extrahepatic lesion (HR 2.47, 95%CI 1.02–5.10, *p* = 0.04) and osteopenia (HR 3.91, 95%CI 2.15–7.07, *p* = 0.001) were associated with OS of colorectal cancer liver metastases (Table 2). In multivariable analysis for OS, osteopenia (HR 3.79, 95%CI 2.09–6.87, *p* = 0.001) was associated with OS in patients with colorectal cancer liver metastases.

**Table 2:** The relationship between patients characteristics and overall survival.

	HR	Univariable	*p* value	HR	Multivariable	*p* value
		95% CI			95% CI	
Age, ≥ 65	1.02	0.99–1.04	0.15			
Gender, Male	0.81	0.50–1.37	0.43			
Lymph node metastases, yes	1.52	0.91–2.63	0.12			
Extrahepatic lesion, yes	2.47	1.02–5.10	0.04	2.02	0.91–4.48	0.08
Timing of tumor, Metachronous	1.03	0.61–1.70	0.93			
Preoperative chemotherapy, no	1.56	0.92–2.61	0.09			
Postoperative chemotherapy, yes	0.86	0.51–1.46	0.57			
Tumor Number, Multiple	1.50	0.92–2.45	0.11			
Stage, III, IV	1.39	0.72–2.66	0.32			
Tumor Size, ≥ 50 cm	1.52	0.84–2.59	0.13			
Osteopenia, yes	3.91	2.15–7.07	0.001	3.79	2.09–6.87	0.001
Sarcopenia (PMI), yes	0.79	0.48–1.36	0.39			
Sarcopenia (IMAC), yes	0.99	0.58–1.80	0.98			
ASA PS, 2 or 3	1.35	0.67–2.48	0.38			
NLR, ≥ 1.93	1.19	0.65–2.17	0.57			
PLR, ≥ 255	0.46	0.11–1.92	0.29			
PNI, ≥ 40	0.68	0.32–1.68	0.38			
CEA, ≥ 4.5 ng/ml	1.59	0.95–2.77	0.09			
CA19−9, ≥ 37 U/ml	1.47	0.88–2.41	0.13			
Curability, R1	1.42	0.70–2.63	0.31			

HR: Hazard Ratio, Confidence interval: CI, PMI: Psoas muscle index, IMAC: Intramuscular adipose tissue, ASA: American Society of Anesthesiologists, PS: Physical status, NLR: Neutrophil to lymphocyte ratio, PLR: Platelet to lymphocyte ratio, PNI: Prognostic nutrient index, CEA: carcinoembryonic antigen, CA: Carbohydrate antigen

### Relationship between recurrence-free survival and patient variables

**Fig 2:**
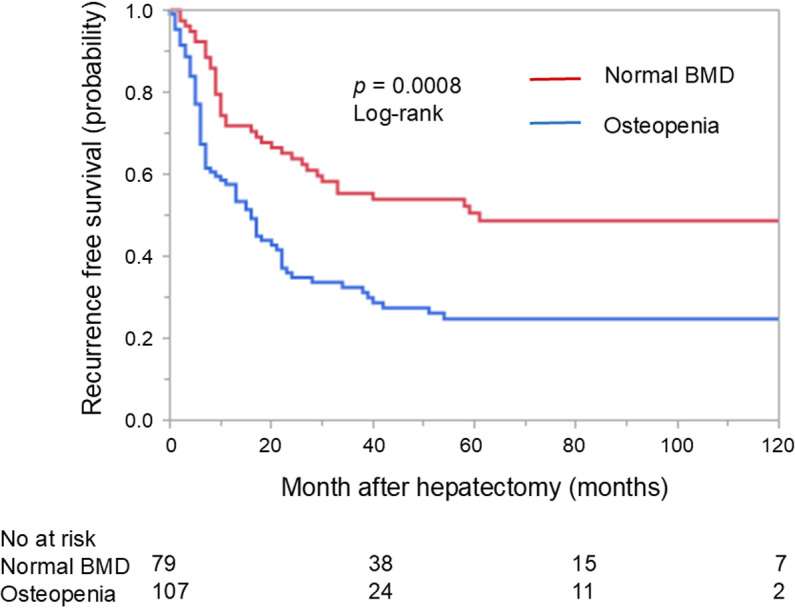
Recurrence-free survival in patients with or without osteopenia.

RFS was significantly shorter in the osteopenia group than the normal BMD group (*p* = 0.0008, 5-year survival: 26.3% vs 51.5%, Fig 2).

In univariable analysis for RFS, pre-operative chemotherapy (HR 2.00, 95%CI 1.38–2.92, *p* = 0.003), tumor number (HR 1.81, 95%CI 1.24–2.64, *p* = 0.002), and osteopenia (HR 1.94, 95%CI 1.31–2.94, *p* = 0.0008) were associated with RFS in colorectal cancer liver metastases patients (Table 3). In multivariable analysis for RFS, preoperative chemotherapy (HR 1.92, 95%CI 1.12–3.30, *p* = 0.002), tumor number (HR 1.51, 95%CI 1.02–2.27, *p* = 0.04), and osteopenia (HR 2.18, 95%CI 1.46–3.24 *p* = 0.001) were associated with RFS in patients with metastases.

**Table 3:** The relationship between patients characteristics and recurrence-free survival.

	HR	Univariable	*p* value	HR	Multivariable	*p* value
		95% CI			95% CI	
Age, < 65y	0.82	0.55–1.20	0.31			
Gender, Male	0.93	0.64–1.38	0.71			
Lymph node metastases, yes	1.45	0.98–2.18	0.07			
Extrahepatic lesion, yes	1.76	0.89–3.15	0.10			
Timing of tumor, Metachronous	1.38	0.95–2.02	0.09			
Preoperative chemotherapy, no	2.00	1.38–2.92	0.0003	1.79	1.20–2.66	0.004
Postoperative chemotherapy, yes	0.72	0.45–1.06	0.10			
Tumor Number, Multiple	1.81	1.24–2.64	0.002	1.51	1.02–2.27	0.04
Stage, III, IV	1.54	0.94–2.53	0.09			
Tumor Size, ≥ 50 cm	1.38	0.87–2.10	0.17			
Osteopenia, yes	1.94	1.31–2.94	0.0008	2.18	1.46–3.24	0.0001
Sarcopenia (PMI), yes	1.10	0.70–1.63	0.70			
Sarcopenia (IMAC), yes	1.33	0.85–2.15	0.22			
ASA PS, 2 0r 3	1.18	0.67–2.06	0.57			
NLR, ≥ 1.93	1.54	0.41–1.01	0.051			
PLR, ≥ 255	2.83	0.89–8.98	0.08			
PNI, ≥ 40	0.78	0.51–1.69	0.51			
CEA ≥ 4.5 ng/ml	1.17	0.80–1.78	0.42			
CA19−9, ≥ 37 U/ml	1.15	0.76–1.70	0.49			
Curability, R1	1.55	0.92–2.46	0.09			

HR: Hazard Ratio, Confidence interval: CI, PMI: Psoas muscle index, IMAC: Intramuscular adipose tissue, ASA: American society anesthesiologists, PS: Physical status NLR: Neutrophil to lymphocyte ratio, PLR: Platelet to lymphocyte ratio, PNI: Prognostic nutrient index, CEA: carcinoembryonic antigen, CA: Carbohydrate antigen

### Recurrence site and association of KRAS mutation and bone mineral density

In multivariable analysis of the association between post-hepatectomy recurrent sites and osteopenia, lung metastasis had about 5 times more frequency for osteopenia patients (OR 5.39, 95%CI 1.83–15.8, *p* = 0.002, Table 4).

**Table 4:** The relationship between secondary metastases site and osteopenia.

	Odds ratio	Univariable	p value	Odds ratio	Multivariable	p value
		95% Confidence interval			95% Confidence interval	
Liver metastases, Yes	1.35	0.72–2.53	0.35	2.00	0.96–4.16	0.07
Lung metastases, Yes	4.02	1.47–11.0	0.007	5.39	1.83–15.8	0.002
The other sites, Yes	0.92	0.50–1.70	0.79	1.47	0.71–3.02	0.29

**Fig 3:**
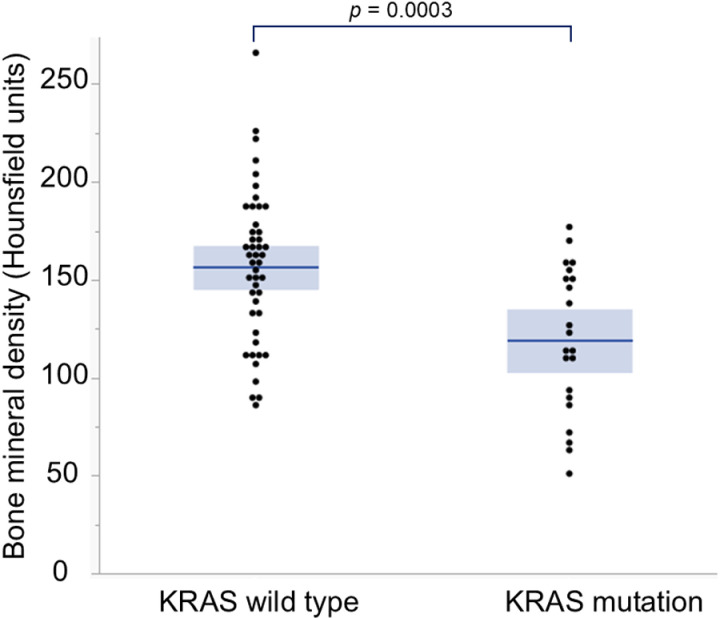
Box-plot of bone mineral density with or without KRAS mutation.

Out of 187 patients with KRAS analysis performed in 68 patients as routine examination, the KRAS mutation patient BMD value was higher than that in patients with KRAS wild type (156 ± 38 vs 118 ± 37HU, *p* = 0.001, Fig 3).

## Discussion

In this study, osteopenia was an independent risk factor for survival and post-hepatectomy recurrence in colorectal liver metastasis patients. Additional recurrence risk factors were postoperative chemotherapy, tumor number, and osteopenia. This study also suggests the importance of assessing preoperative BMD in patients undergoing resection. The mechanism of the association between cancer progression, metastases, and osteopenia remains unclear. Osteopenia patients were found about 5 times more likely to have lung metastases after hepatectomy. Also, the KRAS mutation group tended to have lower BMD than the KRAS wild-type. KRAS mutation in colorectal cancer may be associated with aggressive tumor behavior through increased invasiveness and higher rates of lung metastases [22,23]. Lung metastasis was more common at diagnosis in patients whose primary tumor carried a KRAS mutation and was more likely to develop during the disease course [24]. This suggests a potential metastatic tropism of colorectal cancer to the lung that may have clinical significance for treatment planning.

Studies have shown elevated C-reactive protein, Interleukin(IL)-6, and tumor necrosis factor-alpha levels in frail adults, indicating a potential role of chronic inflammation [25]. Elevated IL-6 levels are associated with advanced tumor stages, increased tumor size, metastasis, and shortened survival of colorectal cancer patients [25]. In many instances, locally produced IL-6 entered the peripheral circulation and caused systemic effects such as cachexia and tumor-associated syndromes (fever, increased erythrocyte sedimentation rate, increased serum C-reactive protein levels, hypoalbuminemia) [26]. When various cytokines that contribute to cancer cachexia such as transforming growth factor-β, IL-6, and tumor necrosis factor-alpha are inhibited, bone mineral density loss is attenuated, most likely by preventing full differentiation of osteoclasts through the receptor activator of nuclear factor-κB receptor activator of nuclear factor-κB ligand (RANKL) pathway [27–29]. Receptor activator of nuclear factor-κB (RANK) gene expression was associated with decreased survival and KRAS mutation [30]. Since cancer and cachexia are essentially pathologies that develop due to inflammation, diverse clinical manifestations of frailty and osteopenia may be associated with a variety of inflammatory cytokines [31]. In general, lung metastasis as a single organ metastasis of colorectal cancer has a better prognosis than metastasis to liver, [32,33] but cases here may be slightly different as lung metastasis follows liver metastasis. Taken together, BMD loss may be associated with KRAS mutation mechanisms through the RANK-RANKL axis and thus frequency of lung metastases.

Ikuta et al have shown that osteopenia was an independent risk factor for worse prognosis but only borderline significance for RFS in colorectal cancer liver metastases [11]. In this study, low BMD was significantly associated with shorter RFS. One explanation for this discrepancy may be that low BMD-specific outcomes, particularly frailty fractures, have a significant negative impact on physical activity and functional status, leading to non-adherence or discontinuation of cancer therapy, or non-cancer mortality [11]. Toshima et al and Sharma et al also showed that bone loss was independently associated with survival after liver transplantation in hepatocellular carcinoma patients, but not with recurrence, [19,34] which indicated that relapse treatment effectiveness may be related to osteopenia [18,35]. Results here also suggest that perioperative treatment has an impact on post-hepatectomy recurrence.

Cancer chemotherapy (e.g., antineoplastic drugs such as platinum-derived com-pounds, alkylating agents, antimetabolites, glucocorticoids, and targeted therapies) accelerates bone loss through direct dysregulation of bone metabolism and other indirect mechanisms. The combination therapy Folfiri (5 fluorouracil, leucovorin, and irinotecan) also reduces bone volume [34]. Here, osteopenia patients had a shorter survival rate than non-osteopenia patients, even though osteopenia patients received chemotherapy at a higher rate. This may be because advanced disease patients with concurrent liver metastases from colorectal cancer are more likely to receive preoperative chemotherapy, and BMD reduction effects due to tumor burden may be greater than due to chemotherapy.

Sarcopenia may be a prognostic factor for various cancers [8,13,36]. Furukawa et al described osteosarcopenia as a strong prognostic predictor in patients with post-hepatectomy colorectal cancer liver metastasis [37]. Many studies have only focused on skeletal muscle mass through CT scan skeletal muscle area assessment. In contrast, insufficient attention has been given to muscle quality deterioration associated with muscle fat deposition [19], despite IMAC’s potential prognostic usefulness [13]. However, in this study sarcopenia and osteosarcopenia were not risk factors for worse OS and RFS outcomes. BMD loss may precede muscle quantity loss, and thus be an early marker of deconditioning preceding quantity or quality muscle loss in end-stage liver disease patients [35,37].

In consideration of pathophysiology, nutritional support and adjunctive therapies such as rehabilitation are important for perioperative bone loss prevention in colorectal cancer liver metastases patients. For pharmacotherapy of osteopenia, vitamin D, calcium, osteoporotic drugs such as teriparatide, denosumab, and bisphosphates, plus exercise may be effective in improving musculoskeletal health [24].

Limitations of this study include its single-site retrospective design. In addition, information bias may have occurred because data were manually abstracted from medical records. There was also potential for unmeasured confounders in the relationship between BMD and cancer status. The cutoff value to diagnose osteopenia was determined by data according to past reports; however, further studies are necessary to determine optimal cutoff values and the results presented should be prospectively validated in a larger, multi-center study.

## Conclusions

In conclusion, osteopenia impacted postoperative long-term outcomes in patients with colorectal cancer liver metastasis. BMD loss was a risk factor for lung metastases in the second recurrence. Measurement of preoperative BMD may be considered a new selection criterion for surgery, follow-up, or chemotherapy in these patients.
